# Seed Inoculation and Foliar Application of Micronutrients Improve the Yield and Quality of Pinto Bean

**DOI:** 10.1002/pei3.70061

**Published:** 2025-06-22

**Authors:** Mansour Taghvaei, Abdolmajid Nikouei, Mahboubeh Fazaeli, Akbar Hemmati, Andrea Mastinu

**Affiliations:** ^1^ Department of Plant Production and Genetics, School of Agriculture Shiraz University Shiraz Iran; ^2^ Pioneer Biotechnologists of Green Science (PBGS) Knowledge‐Based Company, Shiraz University Shiraz Iran; ^3^ Department of Food Science & Technology, School of Agriculture Shiraz University Shiraz Iran; ^4^ Department of Soil, and Water Research Agricultural and Natural Resources Research and Education Center, AREEO Fars Iran; ^5^ Department of Molecular and Translational Medicine University of Brescia Brescia Italy

**Keywords:** agricultural sustainability, antioxidant enzyme activity, biofertilizers, chlorophyll content, micronutrients, nutrient uptake, root colonization, seed quality

## Abstract

To investigate the effect of bio‐fertilizer pretreatment and foliar application of the micronutrients zinc and iron on the yield and yield components of pinto beans ( *Phaseolus vulgaris* L.). The experimental treatments included a control, pretreatment with mycorrhizal fungi, pretreatment with rhizobial bacteria, combined pretreatment with mycorrhizal fungi and rhizobial bacteria, and additional combinations of these with foliar applications: mycorrhizal fungi and rhizobial bacteria with iron foliar spray, with zinc foliar spray, and with both iron and zinc foliar sprays. Bio‐fertilizer pretreatment significantly increased all studied traits compared to the control. The combination of seed inoculation with mycorrhizal fungi and rhizobial bacteria, along with iron foliar application, notably enhanced plant height, root colonization, leaf chlorophyll content, antioxidant enzyme activities, and nutrient levels in seeds, including nitrogen, potassium, and iron. However, the foliar application of zinc and iron resulted in reduced phosphorus content in the seeds, with the highest zinc content observed in the treatment combining bio‐fertilizers with zinc foliar spray. The combined bio‐fertilizer and iron foliar treatment achieved the highest grain yield and biological yield. These results indicate that the use of bio‐fertilizers, along with foliar applications of iron and zinc, can effectively enhance the morpho‐physiological characteristics, yield, and quality of pinto beans.

## 1 Introduction

Pulses, including beans ( *Phaseolus vulgaris* L.), are among the most important sources of plant‐based protein. Beans provide approximately 22% of the protein and fat needed by humans globally, as noted by Ramphisa and Davenport (2020). Their high protein content makes them comparable to meat but with lower fat and cholesterol levels. Globally, beans are cultivated on approximately 27 million hectares, with an average yield of 568 kg per hectare, resulting in a total production of 15.5 million tons. In Iran, bean cultivation spans around 108,000 ha, and the cultivated area is increasing (FAO 2022).

One key advantage of bean cultivation is its ability to stabilize atmospheric nitrogen through symbiotic relationships with *Rhizobial* bacteria. This relationship supplies a significant portion of the nitrogen required by the plants and improves long‐term soil fertility (Hajikhani et al. 2011). However, the overuse of chemical fertilizers and insufficient attention to the benefits of biological fertilizers has led to increased environmental pollution and a gradual decline in soil fertility in Iran (Aslam et al. 2020). Addressing these challenges requires inputs that not only increase nutrient availability but also enhance the physical and microbial properties of the soil (Figueira et al. 2019).

*Rhizobial* bacteria and arbuscular mycorrhizal fungi (AMF) are among the most important bio‐fertilizers in modern agriculture. These growth‐promoting microorganisms play a vital role in supplying nutrients to plants by colonizing their root systems (Adeleke et al. 2021).

Additionally, they can enhance plant growth and nutrient uptake indirectly through the production of phytohormones, antioxidant enzymes such as peroxidase (POD), ascorbate peroxidase (APX), and catalase (CAT), along with other beneficial compounds (Zhu et al. 2010). These bacteria can even absorb nutrients under drought stress (Goswami et al. 2016). AMF form symbiotic relationships with over 80% of crop plants (Frosi et al. 2016), helping plants take up more nutrients under both stress and non‐stress conditions, thereby promoting better growth and yield. AMF can improve nutrient and water uptake in roots, photosynthetic activity, and hydraulic conductivity (Khalaj et al. 2019). One strategy to reduce the ecological impact of agriculture is biological pretreatment or bio‐priming, where seeds are inoculated with beneficial soil microorganisms (Das et al. 2018). For example, inoculating bean seeds with *Rhizobium phaseoli* bacteria has increased plant height, pod length, and leaf nitrogen content (Fallahi and Sharifi 2020). Similarly, studies have shown that using rhizobial bacteria in beans can increase 100‐seed weight, number of pods, and seeds per pod (12Research on soybeans has shown that inoculating seeds with both mycorrhizal fungi and bacteria can increase 1000‐seed weight compared to no inoculation; Samsami et al. 2019). Iron and zinc deficiencies are common in Iranian agricultural soils due to high pH, high lime content, and excessive use of phosphate fertilizers (Sadeghi et al. 2021). In such cases, foliar application of iron and zinc chelates is an effective and economical way to supplement these essential micronutrients. Foliar application of m elements like zinc and iron increases plant resistance to adverse environmental conditions by reducing the negative effects of oxidative damage markers like malondialdehyde levels (Sattar et al. 2022). This method is suitable for delivering low‐consumption nutrients such as zinc and iron because it allows direct absorption by the plant's leaves and aerial parts, preventing the formation of insoluble complexes in aerobic soils (Nasiri et al. 2013). Iron, a crucial cofactor in many oxidation–reduction enzymes, is essential for chlorophyll production (Merlo et al. 2014). Zinc, another essential micronutrient, is involved in tryptophan synthesis (a precursor of auxin), chlorophyll maintenance, leaf senescence, carbohydrate metabolism, and protein synthesis (Sharma et al. 2025). SPAD index is a suitable index for selecting common bean genotypes with higher productivity and adaptability in optimal use of organic and inorganic fertilizers too (Carreño Siqueira et al. 2023).

Research has shown that foliar application of zinc and iron can increase plant height, number of pods per plant, seed weight in plant, and biological yield in chickpea plants (Janmohammadi et al. 2018). It was also reported that foliar spraying of bean plants with zinc sulfate increased zinc concentration in seeds by 19% compared to soil application (Kavian Athar and Aboutalebian 2020). Although the effects of zinc and iron fertilizers have been studied on crops such as wheat, mung bean, and beans, no comparative research has yet examined the impact of seed inoculation with mycorrhizal fungi and rhizobial bacteria on productivity, quality, and nutrient uptake in pinto beans ( *P. vulgaris* L.). Maintaining soil health is crucial for improving crop yield and quality while minimizing the environmental and economic risks associated with fertilizers. Given the importance of enhancing bean seed quality through bio‐priming with environmentally friendly microbes and reducing environmental pollution, as well as the limited information in this area, exploring new approaches to plant nutrition management is essential to improve seed yield and quality. We hypothesize that seed inoculation and foliar spraying during plant growth could enhance pinto bean yield, yield components, and seed quality. Therefore, this experiment was conducted to investigate the effect of bio‐fertilizer pretreatment and foliar application of zinc and iron on pinto beans yield and seed quality.

## 2 Material and Methods

The seeds of the Kosha cultivar were obtained from the Vegetable and Irrigated Pulse Crops Research Center or Pulses Research Department, Karaj, Iran. The experiment was conducted at Eghlid Research Field Station (52°42 E and 30°55 N) and field research of the Faculty of Agriculture Shiraz University (29 36′N, 52 32′E) in a factorial experiment based on a randomized complete block design (RCBD) with four replications in the year 2022 and April 2023. The experimental treatments included seven levels.

1. Control (no inoculation and foliar spraying with distilled water).
2. Seed inoculation with mycorrhizal fungi(F).
3. Seed inoculation with rhizobial bacteria(B).
4. Seed inoculation with a combination of mycorrhizal fungi and rhizobial bacteria(FB).
5. Seed inoculation with a combination of mycorrhizal fungi and rhizobial bacteria + foliar spraying of iron sulfate (FB + Fe).
6. Inoculation of seeds with a combination of mycorrhizal fungi and rhizobial bacteria + foliar spraying of zinc sulfate (FB+ Zn).
7. Seed inoculation with a combination of mycorrhizal fungi and rhizobial bacteria(FB) + foliar spraying of a combination of mycorrhizal fungi and rhizobial bacteria (FB + fFB).

The physicochemical characteristics of the soil used in the experiment are listed in Table 1, but the type of soil was “Fine mixed mesic Typic Xerochreps” based on USDA Soil Taxonomy soil. The average monthly temperature and precipitation data for the weather station in the study region in 2023 are listed in Table 2.

**TABLE 1:** Some of the physical and chemical properties of soil used in the experiment.

Soil texture	OC (%)	K (ppm)	P (ppm)	N (%)	Fe (ppm)	Zn (ppm)	EC (d Sm^−1^)	pH
Loam	0.27	306	10	0.14	4.44	0.14	2.1	8.03

*Note:* OC = organic carbon, K = potassium, phosphorus, *N* = nitrogen, Zn = zinc, EC = electrical conductivity, pH = soil reaction.

**TABLE 2:** The average monthly temperature and precipitation data for the weather station in the study region in 2022.

Month	Temperature (°C)	Precipitation (mm)
April	9.5	0
May	12.4	15.5
June	18.4	0
July	20.5	0
August	20.7	1.5
September	16.7	0
October	12.6	0
November	7	18.5
December	4.8	20
January	−1	100
February	−0.8	39.5
March	6.3	18.5

### 2.1 Sowing Patterns

The crop pattern was ridge and furrow with a spacing of 50 cm, a furrow length of 4 m, each plot contained 4 rows, and the distance between the blocks was 2 m.

### 2.2 Seed Inoculation

Seeds were inoculated with the rhizobia (20 g per kg seed) of species 147 and 177 and mycorrhizal fungus (1 L per 100 kg seed) of species *Funneliformis moseae* produced by Soil and Water Research Institute Iran. Before inoculating, the seeds were washed with sterile distilled water. Gum Arabic (from Merck CO) solution (40% w/v) was used (2 mL/100 g seeds) as an adhesive agent to ensure that the rhizobium inoculants coated the seeds well. Seed inoculation with rhizobia was carried out by Eghlid Research Field Station staff at the farm before planting, and the treated seeds were stored under shade until seeding.

### 2.3 Foliar Application

Zinc sulfate (ZnSO_4_) (from Merck CO) and iron sulfate (FeSO_4_) (from Merck CO) sources (3.5 g per liter) were used for foliar spraying of zinc and iron during the vegetative stage (at 24 and 31 days after sowing [DAS], respectively). We used the backpack sprayer brand Chapin International with a fine double flat jet nozzle at about 2.9 bar operating pressure (Garcia et al. 2023). Manual weeding was carried out to control weeds in the field. Abamectin (0.5 L per 1000 L of water) is used as a pesticide to effectively fight ringworm (*Agrotis segetum*).

### 2.4 Plant Height

Plant height was measured from the ground level to the top of the plant at the pod stage (BBCH code 79) for 10 randomly selected plants in each plot by using a ruler.

### 2.5 Root Colonization Percentage

To determine the extent of root colonization by AM fungi, 1‐cm‐long fresh root segments were stained using the method described by (Phillips and Hayman 1970) with minor modifications. The stained root samples were then observed under a light microscope, and the root colonization percentage was quantified using the grid line intersection method (Giovannetti and Mosse 1980; Vierheilig et al. 1998).

### 2.6 SPAD Index

The SPAD index was measured on the fully expanded third leaf from the top at 10:00–12:00 a.m. by using the SPAD chlorophyll meter (Minolta SPAD‐502 m, Tokyo, Japan).

### 2.7 The Chlorophyll Content

Fresh leaf samples (1 g) were crushed with 15 mL of 80% acetone, and the supernatant was collected after centrifugation at 3000 rpm for 15 min. The extract was then diluted to 25 mL using 80% acetone. The leaf extract absorption was measured using a spectrophotometer (20−120‐UV model, Japan) at 645 and 663 nm.

The chlorophyll content was calculated using the following equation:

$$\begin{array}{c} \text{Chlorophyll content}\;\left(\mathrm{mg}\;g^{-1}\;\mathrm{FW}\right)\\ \;=\left[20.2\;\left(A\right)+8.02\;\left(B\right)\times V/\left(W\times 1000\right)\right] \end{array}$$

where *A* and *B* are absorptions at 645 and 663 nm, respectively; *V* displays the final volume of the extract and acetone; and *W* demonstrates fresh leaf weight.

### 2.8 Enzymatic Antioxidant Activities

Fresh leaf samples (0.5 g) were ground with liquid nitrogen in a mortar; 1 mL of 50 mM phosphate buffer containing 0.5 M EDTA‐polyvinyl and 2% polypyrrolidone was added to the extracted tissue and centrifuged at 14,000× *g* for 20 min at 4°C. The supernatant was used for the determination of ascorbate peroxidase (APX) and catalase (CAT) activities (Jam et al. 2023). APX activity was estimated as described by Nakano and Asada (1981) and Beauchamp and Fridovich (1971).

### 2.9 Yield and Yield Components

To assess the yield and yield components, two square meters were harvested from each plot during the fruit and seed ripening stage (BBCH code 89), avoiding the border effect. The following parameters were measured: number of pods per plant, number of seeds per pod, 100‐seed weight, biological yield, seed yield, and harvest index. Additionally, the nutritional contents of the seeds, including the following elements, were analyzed.

Nitrogen was measured using the method of Novozamsky et al. (1974) as follows:

A 0.5 g sample of ground dry plant material was weighed. The sample was subjected to wet digestion, dialysis was used to dilute the plant digest to avoid overlapping absorbance readings, and the nitrogen concentration was read by spectrophotometer at 410 nm.

Phosphorus was measured using Gianoncelli et al. (2015) method as follows: this method is based on the reaction of phosphate ions with ammonium molybdate in an acidic solution, which forms a yellow complex. This complex then converts to molybdenum blue, usually read spectrophotometrically at a wavelength of about 640–700 nm.

Potassium was measured using Peterson's method as follows: 1 g leaf ash of interest was placed on the heater after adding 5 mL of chloridric acid. When boiling began, the resulting solution was passed through filter paper, and the volume of the samples was brought to 100 mL with double‐distilled water. Then the potassium values were read with a flame photometer (Gheisary et al. 2025).

Iron and zinc were measured using the ash method and via atomic absorption spectroscopy as follows: One gram of leaf‐ashed sample was placed on a heater after adding 5 mL of double‐normal hydrochloric acid. When boiling began, the resulting solution was passed through filter paper, and the volume of the samples was brought to 100 mL with double‐distilled water. The iron and zinc contents were measured by atomic absorption spectrometer (Alfian et al. 2024).

The seed protein percentage was calculated by multiplying the seed nitrogen percentage by 6.25 using the Kjeldahl method (Gosukonda et al. 2020).

The harvest index (HI) was calculated as the ratio of grain weight (12% moisture) to total biomass, and to calculate the biological yield, the topsoil biomass samples were dried in an oven at 60°C for 24 h.

### 2.10 Statistical Analysis

Before the analysis, the normality of the data population was verified using the Kolmogorov–Smirnov and Shapiro–Wilk tests in SPSS (10.0) software. The collected data were analyzed by analysis of variance using ‘SAS’ v. 9.4. The Least Significant Difference (LSD) test was utilized to separate means at *p* ≤ 0.05.

## 3 Result

The ANOVA result showed that the treatments (seed inoculation and foliar application) significantly affected the measured traits.

### 3.1 Plant Height

Seed inoculation with mycorrhizal fungi, rhizobial bacteria, and foliar application significantly impacted plant height (Table 3). Comparing the means, it was observed that the plant height increased with seed inoculation and foliar application of iron sulfate. The highest plant height (93.75 cm) was observed in the treatment combining seed inoculation with mycorrhizal fungi and rhizobial bacteria, along with foliar iron application. The lowest plant height (70.20 cm) was recorded in the control treatment (no seed inoculation and no foliar spraying). Rhizobial seed inoculation with mycorrhizal fungi alone or with rhizobial bacteria alone did not significantly affect plant height, but inoculation of mycorrhizal fungi and rhizobial bacteria seed inoculation had a greater effect on plant height (Table 4). The combined use of seed inoculation with mycorrhizal fungi and rhizobial bacteria, along with foliar iron application, augmented the plant height percentage by 33.54% compared to the control treatment (Table 4).

**TABLE 3:** ANOVA of the effects of seed inoculation and foliar application of zinc and iron on plant height, root colonization percentage, SPAD index, chlorophyll content, peroxidase activity, ascorbate peroxidase, and catalase activity in Pinto Beans ( *Phaseolus vulgaris* L.)

Source of variability	df	PH	RCP	SPAD	CC (u mg^−1^ Protein)	POD (u mg^−1^ Protein)	APX (u mg^−1^ Protein)	CAT (u mg^−1^ Protein)
Block	3	83.02**	19.85**	0.04**	0.104**	0.0002**	0.0002**	0.0008**
T	6	292.21**	1046.90**	175.02**	70.64**	0.016**	0.016**	0.014**
Error	18	48.19	10.52	6.41	0.041	0.00003	0.0003	0.0033
C.V(%)		8.75	7.60		1.75	3.17	3.03	2.25

*Note:* The factors included in the analysis are *R* = replication, T = treatment, PH = plant height, RCP = root colonization percentage, SCMR = SPAD index, CC = chlorophyll content, POD = peroxidase activity, APX = ascorbate peroxidase, CAT = catalase activity.

** and * indicate significance at the 1% and 5% probability levels, respectively, while NS denotes non‐significance.

**TABLE 4:** Effects of the interaction between seed inoculation and foliar application of zinc and iron on plant height, root colonization percentage, SPAD index, chlorophyll content, peroxidase activity, ascorbate peroxidase, and catalase activity in Pinto Beans ( *Phaseolus vulgaris* L.)

	PH	RCP	SPAD	CC (u mg^−1^ Protein)	POD (u mg^−1^ Protein)	APX (u mg^−1^ Protein)	CAT (u mg^−1^ Protein)
N	70.20d ± 5.0	13.25e ± 2.21	32.55e ± 2.32	19.32f ± 0.21	0.18.6f ± 0.004	0.19f ± 0.003	0.42e ± 0.002
F	73.83cc ± 6.4	39.75c ± 2.50	41.88d ± 3.22	24.52e ± 0.26	0.23e ± 0.005	0.23e ± 0.005	0.47d ± 0.001
B	73.16cd ± 5.2	30.25d ± 2.21	45.78c ± 3.17	27.73d ± 0.20	0.27d ± 0.003	0.28d ± 0.002	0.52c ± 0.003
FB	75.83cd ± 3.6	50.25b ± 4.03	53.16b ± 3.8	29.18c ± 0.22	0.29c ± 0.005	0.31c ± 0.005	0.53b ± 0.002
FB + Fe	93.75a ± 2.9	59.00a ± 4.32	61.32a ± 4.01	30.37a ± 0.21	0.31a ± 0.006	0.36a ± 0.004	0.57a ± 0.001
FB + Zn	87.33ab ± 4.3	54.50ab ± 3.87	58.23ab ± 4.21	29.62b ± 0.16	0.30b ± 0.002	0.33b ± 0.003	0.56ab ± 0.004
FB + fFB	86.22ab ± 3.7	55.01ab	59.32ab ± 4.01	29.22b ± 0.17	0.30 ± 0.003	0.35a ± 0.004	56ab ± 0.002

*Note:* The data presented are the means from 3 replications. Means followed by the same letter within each row are not significantly different according to the LSD test (*p* ≤ 0.05).

Abbreviations: APX, ascorbate peroxidase; CAT, catalase activity; CC, chlorophyll content; PH, plant height; POD, peroxidase activity; RCP, root colonization percentage; SPAD, SPAD index.

### 3.2 Root Colonization

Root colonization percentage was significantly affected by seed inoculation with mycorrhizal fungi, rhizobial bacteria, and foliar application (Table 3). Seed inoculation with mycorrhizal fungus and rhizobial bacteria significantly increased the colonization percentage compared to the control treatment. The colonization percentages were 39.75% and 30.25% for seed inoculation with mycorrhizal fungi and rhizobial bacteria, respectively, compared to 13.25% in the control treatment (Table 4). The combined use of seed inoculation with mycorrhizal fungi and rhizobial bacteria, along with foliar iron application, augmented the colonization percentage by 77.55% compared to the control treatment (Table 4). The combined use of seed inoculation with mycorrhizal fungi and rhizobial bacteria, along with foliar iron application, augmented the root colonization percentage by 96.66% compared to the control treatment (Table 4).

### 3.3 SPAD Index

The SPAD index is a non‐destructive method for indirectly measuring the photosynthetic capacity of plants. Seed inoculation with mycorrhizal fungi, rhizobial bacteria, and foliar application significantly affected the SPAD index (Table 3). Seed inoculation with mycorrhizal fungi and rhizobial bacteria significantly increased the SPAD index compared to the control treatment. The SPAD index values were 41.88 and 45.78 for seed inoculation with mycorrhizal fungi and rhizobial bacteria, respectively, compared to 32.55 in the control treatment (Table 4). The combined use of seed inoculation with mycorrhizal fungi and rhizobial bacteria, along with iron foliar spraying, enhanced the colonization percentage by 61.32 compared to the control treatment. For rhizobial bacteria, there was no significant difference with the simultaneous use of seed inoculation with mycorrhizal fungi and rhizobial bacteria and zinc foliar spraying (Table 4). The combined use of seed inoculation with mycorrhizal fungi and rhizobial bacteria, along with foliar iron application, augmented the SPAD index by 88.38% compared to the control treatment (Table 4).

### 3.4 Chlorophyll Content

Our results showed that seed inoculation with mycorrhizal fungi, rhizobial bacteria, and foliar application had a significant effect on chlorophyll content (Table 3). The lowest chlorophyll content was observed in the control treatment (19.23 u mg^−1^ Protein), while the highest was in the treatment with simultaneous seed inoculation with mycorrhizal fungi and rhizobial bacteria, and iron foliar spraying (30.37 u mg^−1^ Protein). There was also no significant difference with the simultaneous use of seed inoculation with fungi and rhizobial bacteria and zinc foliar spraying (Table 4). Chlorophyll content was much more compared not only to the control treatment but also to seed inoculation with mycorrhizal fungi, rhizobial bacteria, and the treatment with simultaneous seed inoculation with mycorrhizal fungi and rhizobial bacteria. The combined use of seed inoculation with mycorrhizal fungi and rhizobial bacteria, along with foliar iron application, augmented the chlorophyll content by 57.19% compared to the control treatment (Table 4).

### 3.5 POD Activity

Seed inoculation with mycorrhizal fungi, rhizobial bacteria, and foliar application significantly affected the POD activity (Table 3). The lowest POD activity was observed in the control treatment (0.14 u mg^−1^ Protein), while the highest was in the treatment with simultaneous seed inoculation with mycorrhizal fungi and rhizobial bacteria and foliar application of iron (0.31u mg^−1^ Protein) (Table 4). The combined use of seed inoculation with mycorrhizal fungi and rhizobial bacteria, along with foliar iron application, augmented the POD activity by 63.16% compared to the control treatment (Table 4).

### 3.6 APX Enzyme

The results obtained revealed that APX was significantly affected by seed inoculation with mycorrhizal fungi, rhizobial bacteria, and foliar application (Table 3). Seed inoculation with mycorrhizal fungi and rhizobial bacteria increased APX compared to the control treatment, but the highest APX was observed in the simultaneous use of seed inoculation with fungi and rhizobial bacteria and foliar application of iron (0.36 u mg^−1^ Protein) (Table 4). The combined use of seed inoculation with mycorrhizal fungi and rhizobial bacteria, along with foliar iron application, augmented the APX percentage by 89.47% compared to the control treatment (Table 4).

### 3.7 CAT Enzyme

Seed inoculation with mycorrhizal fungi, rhizobial bacteria, and foliar application significantly affected CAT activity (Table 3). The lowest CAT activity was observed in the control treatment (0.42 u mg^−1^ Protein), while the highest CAT was seen in the treatment with simultaneous seed inoculation with mycorrhizal fungi and rhizobial bacteria and iron foliar spraying (0.57). There was no significant difference between this treatment and the treatment with simultaneous seed inoculation with mycorrhizal fungi and rhizobial bacteria and zinc foliar spraying (Table 4). Foliar application of iron can also augment CAT activity in plants. The combined use of seed inoculation with mycorrhizal fungi and rhizobial bacteria, along with foliar iron application, augmented the CAT activity by 35.71% compared to the control treatment (Table 4).

### 3.8 Seed Protein Percentage

Seed protein was significantly affected by seed inoculation with mycorrhizal fungi and rhizobial bacteria (Table 5). The highest seed protein in the first and second years was 24.11 and 27.14, respectively, obtained by treatments with simultaneous seed inoculation with mycorrhizal fungi and rhizobial bacteria and iron foliar spray (Table 6). Nonetheless, the combined use of seed inoculation with mycorrhizal fungi and rhizobial bacteria, as well as the use of rhizobial bacteria seed inoculation in the first and second years, increased seed protein from 15.53 and 20.09 in the control treatment to 21.86 and 24.05 respectively(Table 6).

**TABLE 5:** ANOVA of the effect of seed inoculation and foliar application of zinc and iron on the protein, iron, zinc, phosphorus, and potassium in the Pinto Beans ( *Phaseolus vulgaris* L.) seed.

Source of variability	df	P (%)		Fe (%)		Zn (%)		P (%)		K (%)	
		Year 1	Year 2	Year 1	Year 2	Year 1	Year 2	Year 1	Year 2	Year 1	Year 2
Block	3	0.80**	56.65**	0.26**	1.06**	0.06**	3.69**	0.005**	0.17**	0.01**	0.77**
T	5	14.65**	21.50**	15.26**	18.44**	6.15**	9.93**	1.55**	2.14**	0.54**	0.95**
Error	18	0.22	0.44	0.07	0.08	0.03	0.02	0.001	0.002	0.002	0.003
CV (%)		2.22	2.80	2.32	2.05	3.39	3.15	8.98	2.31	4.20	3.49

*Note:* T = treatment, SP = protein, Fe = iron, Zn = zinc, *P* = phosphores, K = potassium.

** and * significant at the 1% probability level, significant at the 5% probability level, and not significant.

**TABLE 6:** Effect of the interaction of seed inoculation and foliar application of zinc and iron on the protein, iron, zinc, phosphorus, and potassium in the seed of Pinto Beans ( *Phaseolus vulgaris* L.) seed.

	P (%)		Fe (%)		Zn (%)		P (%)		K (%)	
	Year 1	Year 2	Year 1	Year 2	Year 1	Year 2	Year 1	Year 2	Year 1	Year 2
N	15.53e ± 0.85	20.09f ± 2.07	8.71e ± 0.16	9.98e ± 0.42	3.66f ± 0.08	2.87f ± 0.69	1.46e ± 0.39	0.58f ± 0.13	0.81f ± 0.06	0.47b ± 0.37
F	19.00e ± 0.46	21.68e ± 2.94	11.12d ± 0.16	12.79d ± 0.45	4.37c ± 0.12	4.78e ± 0.69	2.04c ± 0.02	2.23d ± 0.14	1.00e ± 0.08	1.16e ± 0.27
B	21.09d ± 0.29	23.70d ± 2.51	10.73d ± 0.19	12.51d ± 0.47	4.67d ± 0.17	5.12d ± 0.75	2.01c ± 0.04	1.87e ± 0.19	1.14d ± 0.03	1.31d ± 0.33
FB	21.86 bc ± 0.5	24.05 cd ± 2.9	12.73c ± 0.34	14.60b ± 0.41	5.24c ± 0.19	6.3b ± 0.73	2.29a ± 0.06	2.41bc ± 1.77	1.29c ± 0.04	1.45 ± 0.38
FB + Fe	24.11a ± 0.69	27.14a ± 3.35	14.58a ± 0.27	16.70a ± 0.59	5.67b ± 0.15	6.30b ± 0.67	1.83d ± 0.04	2.79a ± 0.17	1.55a ± 0.07	2.04a ± 0.32
FB + Zn	22.11b ± 0.25	25.05b ± 2.92	13.07bc ± 0.27	14.94b ± 0.31	6.62a ± 0.29	8.07a ± 0.91	1.47e ± 0.07	2.49 b ± 0.15	1.55a ± 0.07	1.70b ± 0.29
FB + fFB	23.12 ± 0.69	24.05 dc ± 3.43	13.17 ± 0.16	13.58c ± 0.55	5.22 ± 0.15	5.26d ± 0.72	2.09 ± 0.029	2.45 bc ± 0.16	1.51 ± 0.08	1.49c ± 0.35

*Note:* For each variable, the mean (SD) values followed by the same letters in each column are not significantly different using LSD at the 5% level. SP = protéine, *P* = phosphores, K = potassium, Zn = zinc, Fe = Iron.

### 3.9 Iron, Zinc, Phosphorus, and Potassium Percentage

The percentages of iron, zinc, phosphorus, and potassium were significantly affected by seed inoculation with mycorrhizal fungi, rhizobial bacteria, and foliar application (Table 5). The highest of iron (14.58, 16.70) and potassium (1.55, 2.04) in the first and second year were observed by treatments with simultaneous seed inoculation with mycorrhizal fungi and rhizobial bacteria and iron foliar spray respectively(Table 6). However, the highest zinc (6.62 and 8.07) in the first and second years were observed by treatments with simultaneous seed inoculation with mycorrhizal fungi and rhizobial bacteria and zinc foliar spray respectively (Table 6). The highest phosphorus potassium (1.55) in the first year was obtained by treatments with simultaneous seed inoculation with mycorrhizal fungi and rhizobial bacteria and iron foliar spray, and the second year was obtained by treatments with simultaneous seed inoculation with mycorrhizal fungi and rhizobial bacteria (Table 6).

### 3.10 Pods per Plant, Grain per Pod, 100 Seed Weight, Grain Yield, Biological Yield, and Harvest Index

Seed inoculation with mycorrhizal fungi, rhizobial bacteria, and foliar application significantly affected pods per plant, grain per pod, 100 seed weight, grain yield, biological yield, and harvest index (Table 7). The highest pods per plant (8.69 and 10.42) in the first and second years were obtained with seed inoculation with mycorrhizal fungi and rhizobial bacteria + foliar application of iron, respectively (Table 8). However, there was no significant difference with treatment with seed inoculation with mycorrhizal fungi and rhizobial bacteria + foliar application of iron. Seed inoculation with rhizobial and mycorrhizal fungi, along with foliar application of iron, increased the number of grains per pod in the first and second years from 3.78 and 3.92 in the control treatment to 4.9 and 5.43, respectively (Table 8). The highest 100‐seed weight (41.57 and 43.60) in the first and second years was obtained with seed inoculation using a combination of mycorrhizal fungi and rhizobial bacteria, along with foliar application of iron. However, this was not significantly different from the treatment with seed inoculation using a combination of mycorrhizal fungi and rhizobial bacteria, along with foliar application of zinc (Table 8). The highest grain yield (5630 and 5237 g) in the first and second years was obtained with seed inoculation using a combination of mycorrhizal fungi and rhizobial bacteria, along with foliar application of iron (Table 8). Seed inoculation with rhizobial and mycorrhizal fungi, along with foliar application of iron, increased the biological yield in the first and second years from 6210and 6216 in the control treatment to 9665 and 8049, respectively (Table 8). However, there was no significant difference in treatment with seed inoculation with mycorrhizal fungi and rhizobial bacteria + foliar application of iron in the first year. The harvest index (56.44 and 57.13)in the first and second years was obtained with seed inoculation using a combination of mycorrhizal fungi and rhizobial bacteria, along with foliar application of iron (Table 8).

**TABLE 7:** ANOVA of the effects of seed inoculation and foliar application of zinc and iron on pods per plant, grain per pod, 100SW, grain yield, biological yield, harvest index in Pinto Beans ( *Phaseolus vulgaris* L.)

Source of variability	df	PP		GP		100SW		GY		BY		HI	
		Year 1	Year 2	Year 1	Year 2	Year 1	Year 2	Year 1	Year 2	Year 1	Year 2	Year 1	Year 2
Block	3	9.4**	5.61**	0.20**	1.35**	22.4**	155.64**	192,895.81**	135,239.14**	232,395.24**	310,917.48**	6.27	15.57nc
T	6	5.31*	6.66*	0.68*	1.044*	6.65*	8.49*	66,391,035.57**	5,572,178.6**	5,319,295.24**	3,664,703.33**	443.86**	336.92**
Error	18	1.80	1.73	0.16	0.20	3.20	3.53	172,532.14	74,056.48	539,295.24	196,733.81	7.55	6.99
CV (%)		16.89	15.47	9.92	10.06	4.56	4.49	10.64	7.09	8.76	5.42	6.03	5.95

Abbreviations: 100SW, 100 seed weight; BY, biological yield; GP, grain per pod; GY, grain yield; HI, harvest index; PP, pods per plant; T, treatment.

**, *, and ns significant at the 1%, significant at the 5% probability level, and not significant.

**TABLE 8:** Effect of interaction of seed inoculation and foliar application of zinc and iron on pods per plant, grain per pod, 100 seed weight, grain yield, biological yield, and harvest index.

	PP		GP		100SW		GY		BY		HI	
	Year 1	Year 2	Year 1	Year 2	Year 1	Year 2	Year 1	Year 2	Year 1	Year 2	Year 1	Year 2
N	6.01c ± 0.41	6.29c ± 0.55	3.78bc ± 0.34	3.92c ± 0.21	36.66b ± 1.81	41.2bc ± 3.19	1815e ± 155	1830 f ± 157.42	6210c ± 469	6216d ± 472.95	29.28e ± 2.27	29.49d ± 2.45
F	8.15ab ± 0.94	8.55ab ± 1.32	4.15bc ± 0.21	4.57bc ± 0.85	39.30ab ± 1.05	41.93bc ± 4.08	2700d ± 360	2720e ± 363.06	8048b ± 496	8114c ± 576.78	33.28e ± 3.26	33.49c ± 3.27
B	7.14bc ± 1.5	7.55bc ± 0.99	4.25b ± 0.49	4.61b ± 0.53	38.70ab ± 1.82	42.23ab ± 3.14	3775c ± 268	3795d ± 265.42	8105b ± 581	8529bc ± 266.6	44.30d ± 2.69	44.48b ± 2.66
FB	7.86abc ± 2.45	8.27b ± 2.82	3.62c ± 0.06	3.94c ± 0.52	39.52b ± 0.81	39.34c ± 6.98	4190c ± 223	4382bc ± 240.22	8520b ± 634	8889ab ± 635.33	49.29c ± 3.92	49.46ab ± 3.95
FB + Fe	8.69a ± 0.85	10.42a ± 1.24	4.90a ± 0.80	5.43a ± 0.51	41.57a ± 1.14	43.60a ± 5.66	5630a ± 863	5237a ± 308.11	9965a ± 634	9166a ± 279.94	56.44a ± 2.27	57.13a ± 2.06
FB + Zn	8.56ab ± 0.91	8.82ab ± 1.62	4.04bc ± 0.17	4.62b ± 0.93	40.23a ± 1.21	43.15ab ± 5.73	4880b ± 386	4209c ± 225.82	8925ab ± 570	8049c ± 508.93	54.08ab ± 1.75	52.49a ± 3.26
FB + fFB	8.28ab ± 0.84	9.06ab ± 0.76	4.30ab ± 0.38	4.34bc ± 0.35	39.70b ± 1.83	40.83 ± 5.12	4362bc ± 240	4662b ± 385.86	8880ab ± 272	8334bc ± 350.38	52.16bc ± 20.04	50.78ab ± 1.77

*Note:* For each variable, the mean (SD) values followed by the same letters in each column are not significantly different using LSD at the 5% level.

Abbreviations: 100SW, 100 seed weight; BY, biological yield; GP, grain per pod; GY, grain yield; HI, harvest index; PP, pods per plant.

## 4 Discussion

Seed inoculation with mycorrhizal fungi and bacteria can enhance plant height through various mechanisms, such as producing compounds that facilitate nutrient absorption, increasing the availability of soil minerals (e.g., nitrogen fixation), synthesizing plant growth regulators like auxins and gibberellins, and producing enzymes that promote plant growth and development. Iron plays a crucial role in chloroplast development, influencing chloroplast size and enhancing photosynthetic output, which ultimately contributes to increased plant height (Khalaj et al. 2019). These results align with our findings. However, iron deficiency can inhibit auxin biosynthesis, potentially reducing internode elongation and plant height (Nasrollahzadeh Asl and Gorbannezhad 2014). Iron is essential for certain dioxygenases that convert tryptophan into auxin. Inoculation with mycorrhizal fungi and rhizobial bacteria significantly increased root colonization percentage, root length, number of root nodules, and root colonization index (Marzban et al. 2014). Additionally, co‐inoculation of crops with these microorganisms can enhance mycorrhizal uptake by root cells colonized with bacteria (Cavender et al. 2003). Seed inoculation significantly raised the SPAD index compared to the control treatment, as nitrogen—a key component of chlorophyll with one nitrogen and four carbon atoms in its rings—was increased due to rhizobial and mycorrhizal inoculation (Amani et al. 2017). These results are consistent with our findings. Studies have shown that foliar iron application increases chlorophyll content in crops like potatoes (Zhang, Yu, et al. 2022; Zhang, Zhang, et al. 2022), supporting our conclusions. Seed inoculation with rhizobial bacteria enhances nitrogen uptake, subsequently boosting chlorophyll synthesis (Amani et al. 2017). Mycorrhizal fungi increase chlorophyll content under various conditions due to improved water status, photosynthetic capacity, and nutrient uptake (Chandrasekaran et al. 2019). Other research shows that applying rhizobial bacteria, whether by seed inoculation or foliar spray, positively affects chlorophyll in common bean varieties (Sara et al. 2013). Zinc and iron sprays can further elevate the chlorophyll index by activating enzymes involved in chlorophyll synthesis, like carbonic dehydrogenase and tryptophan synthetase (Marzban et al. 2014). The increase in leaf chlorophyll index and subsequent vegetative growth stimulation by zinc and iron may compensate for soil nutrient deficiencies (Nasiri et al. 2013). Iron and zinc foliar sprays enhance chlorophyll levels in crops such as wheat (Ramzan et al. 2020). Iron regulates aminoacetylalanine (ALA) formation, a precursor to chlorophyll and heme (Li et al. 2021), while zinc serves as a structural component of proteins and enzymes and a cofactor for development, affecting chlorophyll levels (Mohammadi et al. 2018). Mycorrhizal fungi and rhizobial bacteria can increase CAT activity in the plant. This is likely because mycorrhizal fungi and rhizobial bacteria can alter the production of plant hormones, which may indirectly enhance CAT activity. Seed inoculation with mycorrhizal fungi and rhizobial bacteria also significantly increased POD activity. Similar effects on POD by seed inoculation have been reported (Sharma and Sharma 2017). Mycorrhizal fungi may enhance POD indirectly by promoting growth, nutrient uptake, and stress tolerance (Zhu et al. 2010). Foliar iron applications also enhance POD content in pea plants due to iron's role as a POD cofactor (Besford and Deen 1977).

## 5 Conclusions

This study demonstrates that seed pretreatment with mycorrhizal fungi and rhizobial bacteria significantly impacts protein and nutrient content and pinto beans' physiological, morphological, and yield components. The combined use of bio‐fertilizers and foliar zinc and iron sprays led to even greater improvements. In particular, foliar iron application, along with bio‐fertilizers, had the most pronounced effect on seed quality and yield. Given the limited availability of iron and zinc in arid and semi‐arid soils, foliar application of these micronutrients is an effective strategy to enhance their concentrations within the plant.

## Conflicts of Interest

The authors declare no conflicts of interest.

## Data Availability

All data are available by connecting the first author through e‐mail address. Furthermore, the extra data and materials used in this study will be made available by the authors upon request as much as possible.
